# Identification of Functional Networks of Estrogen- and c-Myc-Responsive Genes and Their Relationship to Response to Tamoxifen Therapy in Breast Cancer

**DOI:** 10.1371/journal.pone.0002987

**Published:** 2008-08-20

**Authors:** Elizabeth A. Musgrove, C. Marcelo Sergio, Sherene Loi, Claire K. Inman, Luke R. Anderson, M. Chehani Alles, Mark Pinese, C. Elizabeth Caldon, Judith Schütte, Margaret Gardiner-Garden, Christopher J. Ormandy, Grant McArthur, Alison J. Butt, Robert L. Sutherland

**Affiliations:** 1 Cancer Research Program, Garvan Institute of Medical Research, Sydney, Australia; 2 St Vincent's Clinical School, Faculty of Medicine, University of New South Wales (NSW), Sydney, Australia; 3 Peter MacCallum Cancer Centre, East Melbourne, Australia; 4 Department of Medicine, St Vincent's Hospital, The University of Melbourne, Victoria, Australia; University of Birmingham, United Kingdom

## Abstract

**Background:**

Estrogen is a pivotal regulator of cell proliferation in the normal breast and breast cancer. Endocrine therapies targeting the estrogen receptor are effective in breast cancer, but their success is limited by intrinsic and acquired resistance.

**Methodology/Principal Findings:**

With the goal of gaining mechanistic insights into estrogen action and endocrine resistance, we classified estrogen-regulated genes by function, and determined the relationship between functionally-related genesets and the response to tamoxifen in breast cancer patients. Estrogen-responsive genes were identified by transcript profiling of MCF-7 breast cancer cells. Pathway analysis based on functional annotation of these estrogen-regulated genes identified gene signatures with known or predicted roles in cell cycle control, cell growth (i.e. ribosome biogenesis and protein synthesis), cell death/survival signaling and transcriptional regulation. Since inducible expression of c-Myc in antiestrogen-arrested cells can recapitulate many of the effects of estrogen on molecular endpoints related to cell cycle progression, the estrogen-regulated genes that were also targets of c-Myc were identified using cells inducibly expressing c-Myc. Selected genes classified as estrogen and c-Myc targets displayed similar levels of regulation by estrogen and c-Myc and were not estrogen-regulated in the presence of siMyc. Genes regulated by c-Myc accounted for 50% of all acutely estrogen-regulated genes but comprised 85% (110/129 genes) in the cell growth signature. siRNA-mediated inhibition of c-Myc induction impaired estrogen regulation of ribosome biogenesis and protein synthesis, consistent with the prediction that estrogen regulates cell growth principally via c-Myc. The ‘cell cycle’, ‘cell growth’ and ‘cell death’ gene signatures each identified patients with an attenuated response in a cohort of 246 tamoxifen-treated patients. In multivariate analysis the cell death signature was predictive independent of the cell cycle and cell growth signatures.

**Conclusions/Significance:**

These functionally-based gene signatures can stratify patients treated with tamoxifen into groups with differing outcome, and potentially identify distinct mechanisms of tamoxifen resistance.

## Introduction

Among several advances that have contributed to the decreased mortality from breast cancer observed in the past decade, the routine use of adjuvant endocrine therapies directed at the estrogen-estrogen receptor (ER) pathway is a major contributor [1], [2]. Tamoxifen, which blocks estrogen action at its receptor, increases survival following a diagnosis of breast cancer and prevents the development of breast cancer in high risk women [1]–[5]. The more recently-developed aromatase inhibitors, which block estrogen synthesis, appear to be even more effective therapies [6]. Thus, targeting the estrogen receptor pathway is a validated, effective, biologically-based therapy for breast cancer. However, the overall success of this therapeutic approach is limited by both intrinsic and acquired resistance. A significant proportion of patients with ER-positive tumors do not have sustained objective responses, and many who do initially respond subsequently relapse due to the acquisition of endocrine resistance [7]–[9]. Prospective identification of patients who are not good candidates for adjuvant endocrine therapy would substantially facilitate clinical decision-making. To address this need, several gene expression signatures that cosegregate with poor outcome in tamoxifen-treated breast cancer have been derived using gene expression profiling, prospectively-selected candidate genes or differentially-expressed estrogen-regulated genes [reviewed in 10]. A gene expression grade index (GGI) developed as a molecular correlate of histological grade also cosegregates with poor response to tamoxifen therapy [11]. There is little overlap between the genes contained within these signatures, other than the frequent inclusion of genes involved in cell proliferation, and thus although potentially clinically useful, they offer limited insight into the molecular basis of endocrine resistance.

The biochemical and molecular basis of antiestrogen (tamoxifen) resistance has been the subject of intense investigation. Aberrations in ER expression and function, alterations in coactivator and corepressor expression, ligand-independent activation of ER via growth factor-mediated phosphorylation events, a switch from estrogen-driven cell-proliferation to EGFR/erbB2-driven proliferation and the overexpression of various signaling molecules, particularly the mitogen-activated protein kinases and various isoforms of protein kinase C, have all been implicated in endocrine resistance [7]–[9]. Consistent with the idea that deregulation of estrogen target genes, particularly those that mediate cell proliferation and survival, is another potential mechanism of endocrine resistance, overexpression of the estrogen-targeted cell cycle regulatory molecules c-Myc and cyclin D1, which occurs at high frequency in the clinical setting, has been associated with altered sensitivity to endocrine therapy [reviewed in Ref. 12]. Inducible expression of these genes can over-ride antiestrogen-induced growth arrest [13] and overexpression can modulate sensitivity to clinically-relevant antiestrogens in *in vitro* models [reviewed in Ref. 12].

Since estrogen is a multifunctional hormone, we reasoned that the approach of seeking to identify a minimal gene set associated with adverse outcome in tamoxifen-treated patients and the binary nature of many of the resulting classifications might obscure some of the complexity of the underlying biology. Furthermore, several of the endocrine response signatures have been derived using hierarchical clustering, which may not consistently result in stable classification in independent sample sets [14]. With the goal of gaining further mechanistic insights into estrogen action and therefore into endocrine resistance, we sought to classify estrogen-regulated genes by function, and then determine the impact of deregulation of distinct functionally-related sets of genes on the response to tamoxifen in breast cancer patients.

## Results

### Gene expression profiling and identification of estrogen-regulated genes that are also c-Myc-regulated

**Figure 1:**
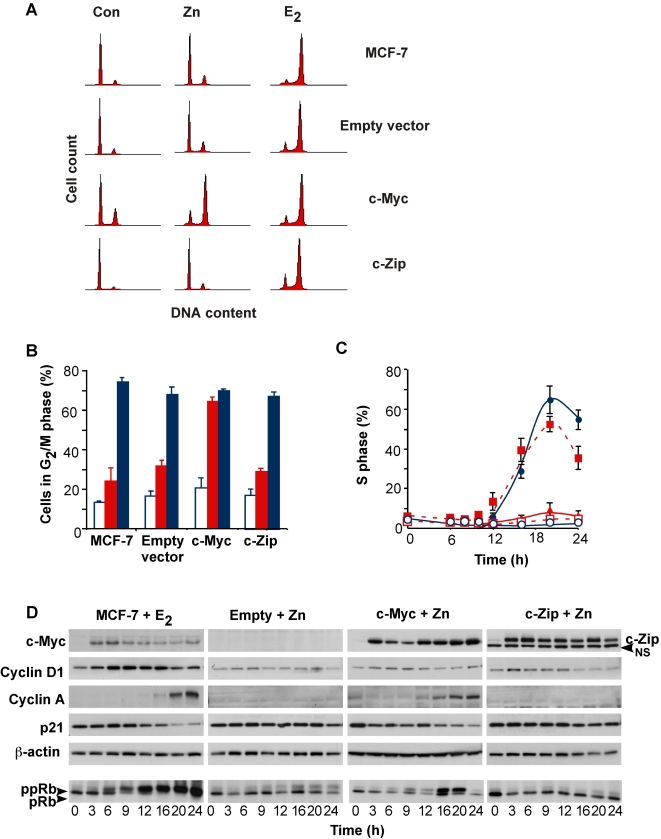
Effects of estrogen treatment and induction of c-Myc and c-Zip in antiestrogen-arrested cells. Cell lines stably transfected with the inducible vector pΔMT containing c-Myc, a c-Myc mutant lacking the entire N-terminal domain (c-Zip) or empty vector (Empty vector) were growth arrested with 10 nM ICI 182780 for 48 h. Cells were treated at time zero with either 100 nM 17β-estradiol (E_2_) or vehicle (ethanol, EtOH) for the parental MCF-7, and 65 µM zinc for empty vector, c-Myc and c-Zip. (A, B) Cells additionally treated with nocodazole to prevent cell division of estrogen-stimulated cells were harvested for analysis of cell cycle phase distribution by flow cytometry. A: Representative histograms 36 h after E_2_ and nocodazole treatment; B: mean±SD of 3 independent experiments. Control (EtOH): open bars; Zn (red) or E_2_ (blue). (C) The proportion of cells in S phase was determined by flow cytometry at intervals after E_2_ or Zn treatment. Data are mean±SD of 3 independent experiments. E_2_, • filled circles; c-Myc, ▪ filled squares; c-Zip, ▴ filled triangles; EtOH, ○ open circles; Empty vector, □ open squares. (D) Cell lysates were analysed by immunoblotting for the proteins shown. Arrowhead indicates a non-specific protein (NS).

Since inducible expression of c-Myc can overcome the inhibitory effects of antiestrogens and recapitulate many of the effects of estrogen on molecular endpoints related to cell cycle progression [13] we reasoned that determining which estrogen-regulated genes were also targets of c-Myc might provide insights into the role of c-Myc in different aspects of estrogen action and in antiestrogen resistance. To this end, a series of clonal MCF-7 cell lines was developed that inducibly express c-Myc or c-Zip (a deletion mutant of c-Myc that lacks the N-terminal transactivation domains). Representative clones that had 17β-estradiol (E_2_) and antiestrogen responses matched to those of the parental MCF-7 cells were chosen for further experiments (Fig. 1A&B). Zinc treatment resulted in increased c-Myc or c-Zip expression within 3 h, similar to the timing of E_2_ induction of c-Myc (see Fig. 1D). Induction of c-Myc led to re-initiation of cell cycle progression and regulation of molecular endpoints that mimicked the effects of E_2_, but induction of c-Zip was ineffective (Fig. 1). Cyclin D1 induction preceded S phase entry in E_2_-treated cells, but was not apparent in zinc-treated control or c-Myc transfected cells (Fig. 1D), consistent with previous data obtained using this model system [13].

**Figure 2:**
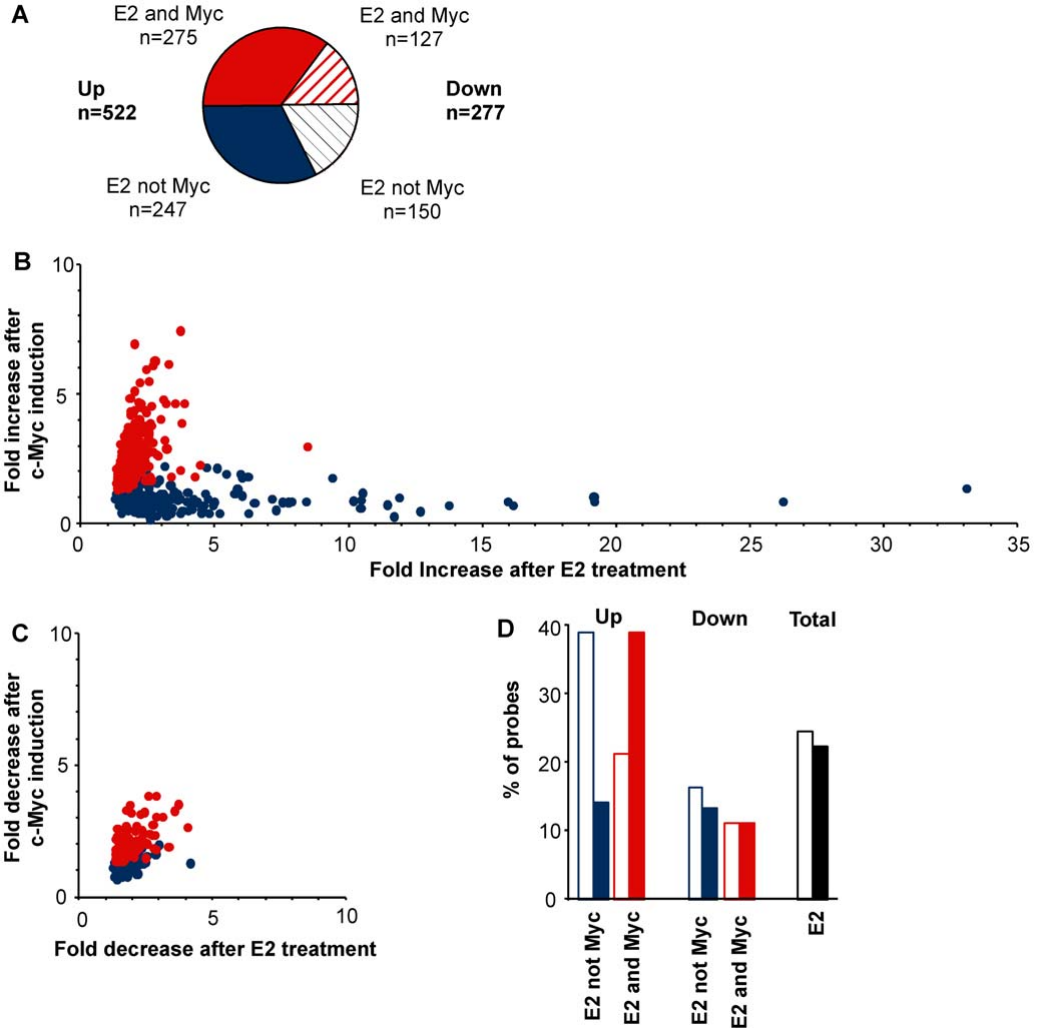
Comparison of genes regulated by estrogen and c-Myc. The experimental design is described in Fig. 1, except that a concentration of 75 µM Zn was used. After 6 h E_2_ or Zn treatment, cells were harvested and transcript profiling undertaken using Affymetrix Human Genome U133 Plus 2.0 oligonucleotide microarrays. After normalisation and correction for multiple hypothesis testing, probesets that were significantly regulated in the same direction by estrogen or c-Myc (but not by c-Zip) were identified (adjusted *P*<0.01). (A) The number of up-regulated (filled) or down-regulated (hatched) genes classified as ‘E_2_ and Myc’ (red) or ‘E_2_ not Myc’ (blue) is shown. (B, C) The fold change in the expression of significantly-regulated probesets following estrogen treatment (relative to vehicle (EtOH) treatment) is shown compared with that following zinc induction of c-Myc (relative to zinc treatment of empty vector cells) as the average of three independent experiments. Red: ‘E_2_ and Myc’; Blue: ‘E_2_ not Myc’. (D) The overlap between probesets in the indicated categories and publically available databases of estrogen-regulated genes (open bars) and c-Myc-regulated genes (filled bars) is shown.

Using mitotically-selected cells, we previously established that MCF-7 cell cycle progression is antiestrogen-sensitive in early-to-mid G_1_ phase, but becomes independent of estrogen signaling 3–4 h before S phase entry [15]. We therefore selected a timepoint within the window of estrogen-dependent cell cycle progression, 6 h after estrogen treatment, and compared the gene expression profile generated after estrogen treatment with that following zinc induction of c-Myc or c-Zip. Initially, genes that were significantly up- or down-regulated following E_2_ treatment compared with vehicle-treated cells were identified (n = 799 genes, represented by 939 probesets, adjusted p<0.01 i.e. false discovery rate 1%). The estrogen-regulated genes were then divided into those that were regulated in the same direction following c-Myc induction or E_2_ treatment but not regulated by c-Zip induction, designated ‘E_2_ and Myc’ (adjusted p<0.01, 402/799 genes i.e. 50%), and the remainder, designated ‘E_2_ not Myc’ (Fig. 2A, Table S1).

The relationship between the response to E_2_ treatment or c-Myc induction for the 635 E_2_-upregulated probesets is shown in Fig. 2B. Those in the ‘E_2_ and Myc’ category formed a cluster which was largely distinct from the cluster designated ‘E_2_ not Myc’. At the zinc concentration used for the microarray experiment, c-Myc expression after zinc induction was higher than after estrogen treatment, and consequently genes in the ‘E_2_ and Myc’ category were more strongly regulated by c-Myc than by estrogen. The ‘E_2_ not Myc’ cluster included the most highly-regulated probes and had an average relative expression of 0.97 after c-Myc induction. In contrast, the distribution of downregulated probes in the ‘E_2_ not Myc’ category essentially overlapped with that of the probes in the ‘E_2_ and Myc’ category (Fig. 2C). Databases of estrogen-responsive genes [16] and c-Myc targets [17] were used to give an indication of the proportion of the genes in each category that had been previously identified as either estrogen- or c-Myc-regulated. Almost 40% of the probes from the ‘E_2_ not Myc’ category that increased in expression were previously-documented estrogen targets (Fig. 2D), significantly more than the corresponding ‘E_2_ and Myc’ probes (*P* = 9.86×10^−7^, Fisher's exact test). A similarly high proportion of the probes in the ‘E_2_ and Myc’ category that increased in expression were present in the Myc target gene database (Fig. 2D), significantly more than in the ‘E_2_ not Myc’ category (*P* = 1.64×10^−12^), suggesting that this analysis identified many *bona fide* c-Myc targets that have not been previously described as estrogen targets. The presence of known c-Myc targets in the ‘E_2_ not Myc’ categories might result, in part, from misclassification, but likely also reflects cell-type specificity in the response to c-Myc induction. For example, *CCND1* (cyclin D1), which is among the genes in the ‘E_2_ not Myc’ category, is present in the c-Myc target gene database but does not increase after c-Myc induction in this experimental model ([13], see also Fig. 1D).

**Figure 3:**
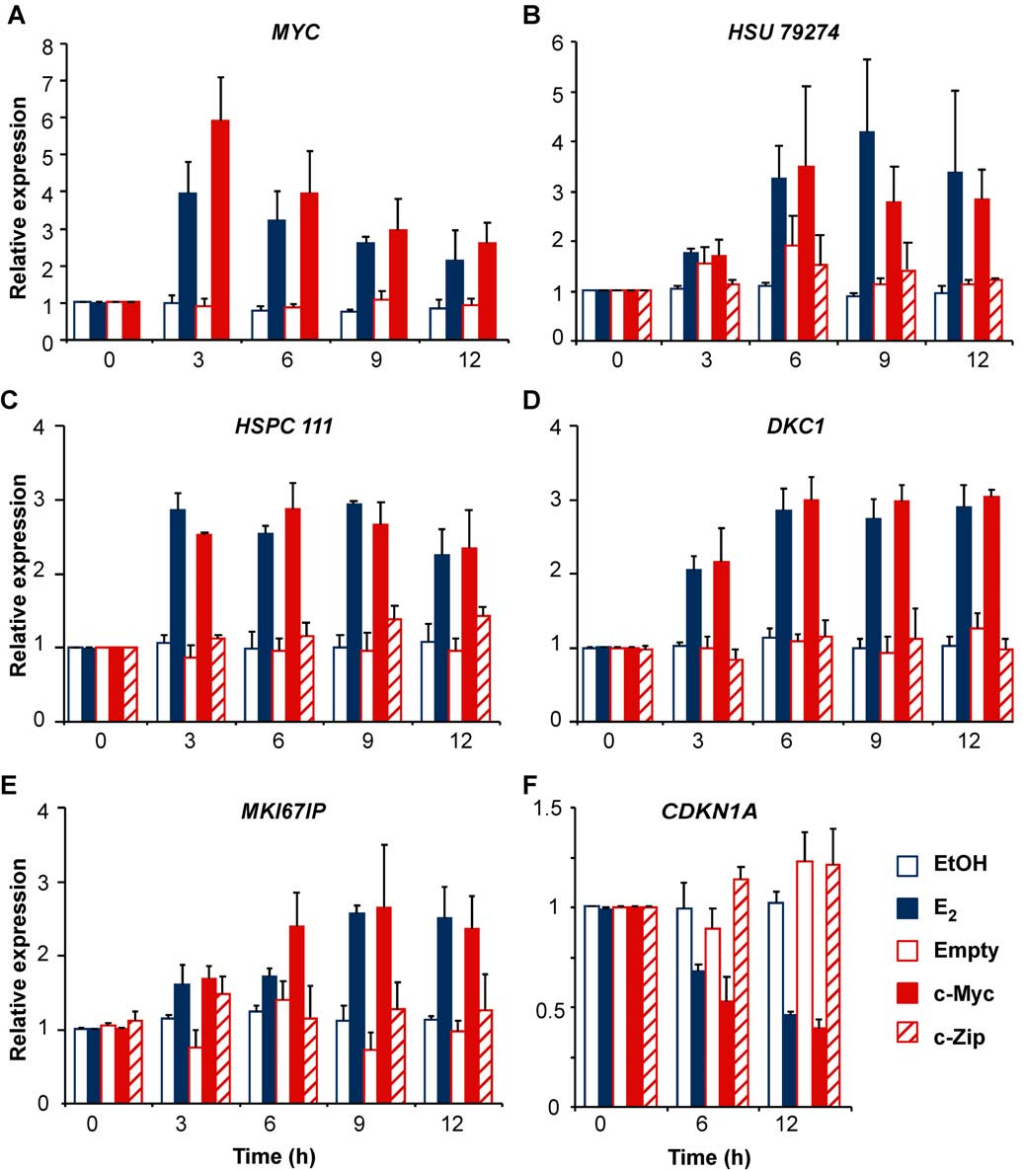
Estrogen and c-Myc regulation of selected genes. The experimental design is described in Fig. 1. For the indicated genes, mRNA levels were quantitated by qRT-PCR and are presented as the mean±range or SEM of 2–3 experiments. EtOH, E_2_, Empty and c-Myc data in (C) are redrawn from [18].

**Figure 4:**
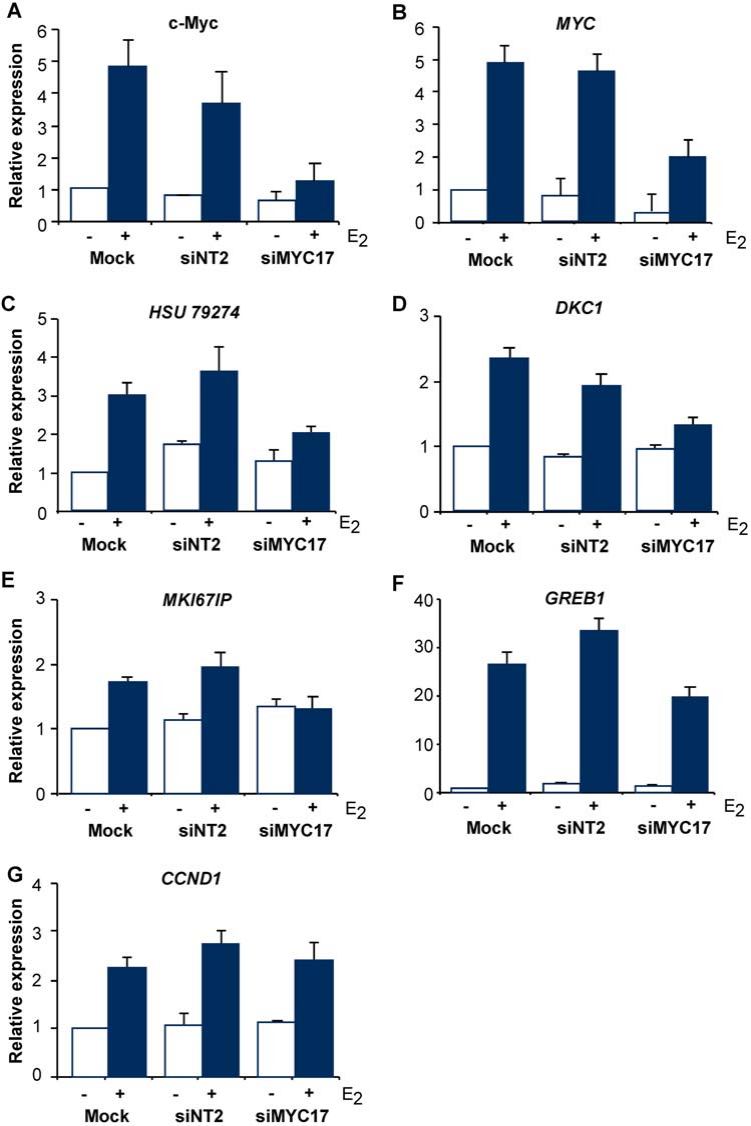
Effect of RNAi-mediated knockdown of c-Myc on regulation of selected genes after estrogen treatment. Cells were mock-transfected or transfected with 50 nM of control (non-targetting siRNA, siNT2) or Myc siRNA (siMYC17), treated with antiestrogen (ICI 182780) for 48 h, and then treated with E_2_ for 6 h (A) or 9 h (B–G). (A) Protein lysates were immunoblotted for c-Myc 6 h after E_2_ treatment and quantitated by densitometry. Data are the mean±range or SEM of 2–3 experiments. (B–G) For the indicated genes, mRNA levels were quantitated by qRT-PCR and are presented as the mean±SEM of 3 experiments.

If a c-Myc-dependent pathway is an integral part of the response to estrogen, the changes in expression of targets in the ‘E_2_ and Myc’ category after estrogen treatment or zinc induction would be expected to be of similar magnitude, provided similar levels of c-Myc were achieved. To test this prediction, we adjusted the concentration of zinc so that the induction of c-Myc mRNA was similar to that after estrogen treatment (Fig. 3A), and examined the expression of selected genes from the ‘E_2_ and Myc’ category. The 5 genes examined all either increased (*HSU79274, HSPC 111, DKC1, MKI67IP*), or decreased (*CDKN1A*, encoding the CDK inhibitor p21^WAF1/Cip1^) in expression to a similar degree after E_2_ treatment or zinc induction of c-Myc (Fig. 2B–F). As a further test of the conclusion that these genes are estrogen regulated via an estrogen-mediated increase in c-Myc expression, MCF-7 human breast cancer cells were stimulated with E_2_ in the presence of siRNAs directed at c-Myc (siMyc). The most effective of the siRNAs tested, siMyc-17, reduced the estrogen induction of c-Myc protein and mRNA at 6–9 h from ∼5-fold to a statistically non-significant level of <2-fold (Fig. 4A&B and data not shown). In the presence of siMyc, none of the genes from the ‘E_2_ and Myc’ category tested (*HSU79274, DKC1, MKI67IP*), displayed significant induction after E_2_ treatment (Fig. 4C–E). In contrast two genes from the ‘E_2_ not Myc’ category, *GREB1* and *CCND1* (encoding cyclin D1), were both significantly induced by E_2_ in either the presence or absence of siMyc (Fig. 4F&G), despite evidence that *CCND1* is a Myc target in other systems. More detailed examination of the regulation of *HSPC 111* showed that its induction by estrogen required ongoing protein synthesis, did not occur in the presence of siMyc, and was accompanied by recruitment of c-Myc to the *HSPC 111* promoter [18]. In addition, others have shown that c-Myc is required for estrogen-mediated decreases in *CDKN1A* expression [19]. Thus, estrogen regulation of all of these 5 genes from the ‘E_2_ and Myc’ category is dependent on c-Myc. Together these data provide strong evidence that our analysis reliably distinguished genes regulated by ‘E_2_ and Myc’ from those regulated by ‘E_2_ not Myc’, and that genes in the ‘E_2_ and Myc ‘ category are regulated via estrogen induction of c-Myc.

### Pathway analysis of estrogen- and c-Myc-regulated genes

**Figure 5:**
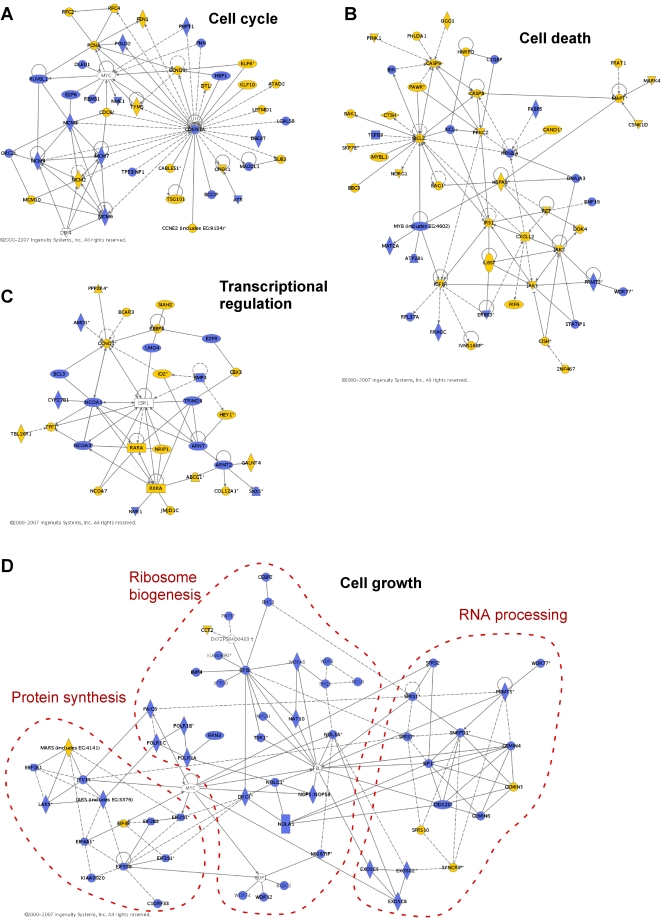
Networks of estrogen-regulated genes. Gene ontology, Ingenuity Pathways analysis and HiMAP were used as detailed in the text to generate networks of estrogen-regulated genes identified by transcript profiling after 6 h estrogen treatment of antiestrogen-arrested cells. Yellow: ‘E_2_ not Myc’; blue: ‘E_2_ and Myc’. Some genes were included for the purposes of illustration e.g. *ESR1*, encoding the ER, and are not coloured. A: cell cycle; B: cell death; C: transcriptional regulation; D: cell growth.

In order to develop hypotheses about the biological processes regulated by estrogen in this system we undertook further analysis using the gene ontology tool Onto-Express [20] and Ingenuity Pathways Analysis, which uses a curated database of known functional interactions to identify networks of mammalian genes. The entire set of estrogen-regulated genes, i.e. both ‘E_2_ and Myc’ and ‘E_2_ not Myc’, contained a significant over-representation of genes in gene ontology biological process categories related to ribosome biogenesis, the cell cycle and cell death (Table 1). Ingenuity analysis of this geneset identified 4 networks with high scores for relevance to the input dataset. These had the following functional annotations: cancer, cell cycle, DNA replication, gene expression and cell death. One network consisted of genes with roles in the cell cycle and its gene signature was expanded by including estrogen-regulated genes from gene ontology categories related to the cell cycle and DNA replication (Fig. 5A, Table S2). Ingenuity analysis of the ‘E_2_ not Myc’ signature identified one high-scoring network that contained genes with roles in cell death and substantially overlapped with two of the high-scoring networks identified using the entire set of estrogen-regulated genes. The main clusters of the latter networks were therefore combined, and the resulting signature expanded by addition of genes from gene ontology categories related to cell death (Fig. 5B, Table S2). The fourth Ingenuity network derived from the entire estrogen-regulated geneset, consisting of genes with roles in transcriptional regulation, was not further modified (Fig. 5C, Table S2).

**Table 1:** Gene ontology of all estrogen-regulated genes.

GO ID	Function Name	Unique UniGene Total	Unique Reference UniGene Total	Corrected P-Value
GO:0006364	rRNA processing	13	37	1.79E-09
GO:0007046	ribosome biogenesis	7	15	1.39E-08
GO:0008033	tRNA processing	9	37	2.69E-06
GO:0006412	protein biosynthesis	25	260	1.34E-04
GO:0006260	DNA replication	17	97	7.97E-07
GO:0006270	DNA replication initiation	6	19	1.75E-05
GO:0048015	phosphoinositide-mediated signaling	4	16	1.63E-03
GO:0006164	purine nucleotide biosynthesis	4	11	1.03E-03
GO:0009396	folic acid and derivative biosynthesis	3	7	1.66E-03
GO:0009113	purine base biosynthesis	3	4	2.58E-03
GO:0008285	negative regulation of cell proliferation	14	134	3.17E-03
GO:0000079	regulation of cyclin dependent protein kinase activity	5	35	6.36E-03
GO:0000074	regulation of cell cycle	18	213	7.49E-03
GO:0008632	apoptotic program	3	9	8.61E-03
GO:0007264	small GTPase mediated signal transduction	20	199	7.22E-04
GO:0042493	response to drug	3	15	9.18E-03

The analysis used Onto-Express, with a binomial distribution model and false discovery rate (FDR) correction for multiple comparisons. Only categories with 3 or more genes are presented, and categories with related functions have been grouped together.

The functional annotation of the final gene signatures was confirmed using Ingenuity (Table S3). The annotation of the ‘cell cycle’ signature revealed a highly significant overrepresentation of genes involved in: DNA replication and DNA metabolism, e.g. several MCMs, PCNA, RFC2 and RFC4; cell cycle control, e.g. *CCND1* and *CCNE2*, encoding cyclins D1 and E2, respectively, and *CDKN1A,* the gene encoding p21^Waf1/Cip1^, which forms a central node in this network; and cancer, including breast cancer. The ‘cell death’ signature was annotated as containing genes involved in cell death, apoptosis and survival. Cancer was also over-represented in this network. Interestingly, for this signature the functional annotations within cancer included invasion and migration as well as cell death and general oncogenic processes (Table S3), and cell movement was significantly overrepresented overall. The ‘transcriptional regulation’ signature included a number of nuclear receptor coregulators, and many of the genes have documented functional interactions with the estrogen receptor (*ESR1*) (Fig. 5C). The annotation of this network identified transcription and cancer as significantly over-represented, as was development of the mammary alveolus, albeit with a small number of genes (Table S3).

The gene ontology classifications of estrogen-regulated genes and the processes represented by the Ingenuity networks displayed many overlapping categories. However, several processes linked by their importance in cell growth, i.e. the increase in cell mass that accompanies progress through the cell cycle, were amongst those most significantly over-represented in the gene ontology (Table 1) but were not well-represented in the Ingenuity networks, i.e. rRNA processing, ribosome biogenesis and protein biosynthesis. This was likely because much of the current understanding of these processes is based on studies in model systems other than mammalian cells. We therefore extended our analysis by predicting likely interactions using the Human Interactome Map (HiMAP), which builds networks based on known protein-protein interactions in human cells and on predictions from interactions in model organisms including yeast, co-ordinate expression in a panel of human tissue samples, shared biological function and conserved protein-protein interaction domains [21]. A gene signature for ‘cell growth’ was compiled from an initial small subnetwork identified by Ingenuity, together with genes that were in relevant gene ontology categories or encoded proteins that proteomic analysis has assigned to the nucleolus [22], [23], and estrogen-regulated genes that were predicted by HiMAP to have direct connections to these genes. The network generated by HiMAP from this list of genes was redrawn using Ingenuity (Fig. 5D) and contained three major clusters that were largely composed of predicted, rather than known, interactions. Using the gene ontology classifications, data from proteomic analysis of the human ribosome biogenesis pathway [23] and annotation of the final network using Ingenuity, these clusters were identified as genes involved in protein synthesis or RNA post-transcriptional modification, particularly splicing, and components of the 90S pre-ribosomal complex (Tables S2 & S3), consistent with the HiMAP prediction of functionally relevant interactions between these genes.

To gain potential insights into the role of c-Myc in estrogen-responsive biological processes, we determined what fraction of the genes in each signature was regulated by c-Myc in our experimental model (Fig. 5, Table S2). In total the ‘cell cycle’ gene signature contained 60 genes, of which 27 (45%) were regulated by both estrogen and c-Myc. Similarly, of the ‘transcriptional regulation’ signature 12 of 31 genes (39%) were regulated by c-Myc. The proportion of genes in these two networks regulated by estrogen and c-Myc was comparable with the 50% overall proportion (*P* = 0.42 and 0.20, respectively, Fisher's exact test). However, genes regulated by both estrogen and c-Myc comprised only one-third of the total genes in the ‘cell death’ network (19/55, i.e. 35%), significantly different from the overall proportion (*P* = 0.017). Conversely, the ‘cell growth’ gene signature contained significantly more genes regulated by c-Myc (102/123, 83%, *P* = 5.5×10^−16^). These data suggest that the degree to which estrogen regulation of cell cycle, cell growth and cell death is secondary to the induction of c-Myc varies significantly.

### Role of c-Myc induction in estrogen stimulation of cell cycle progression and cell growth

**Figure 6:**
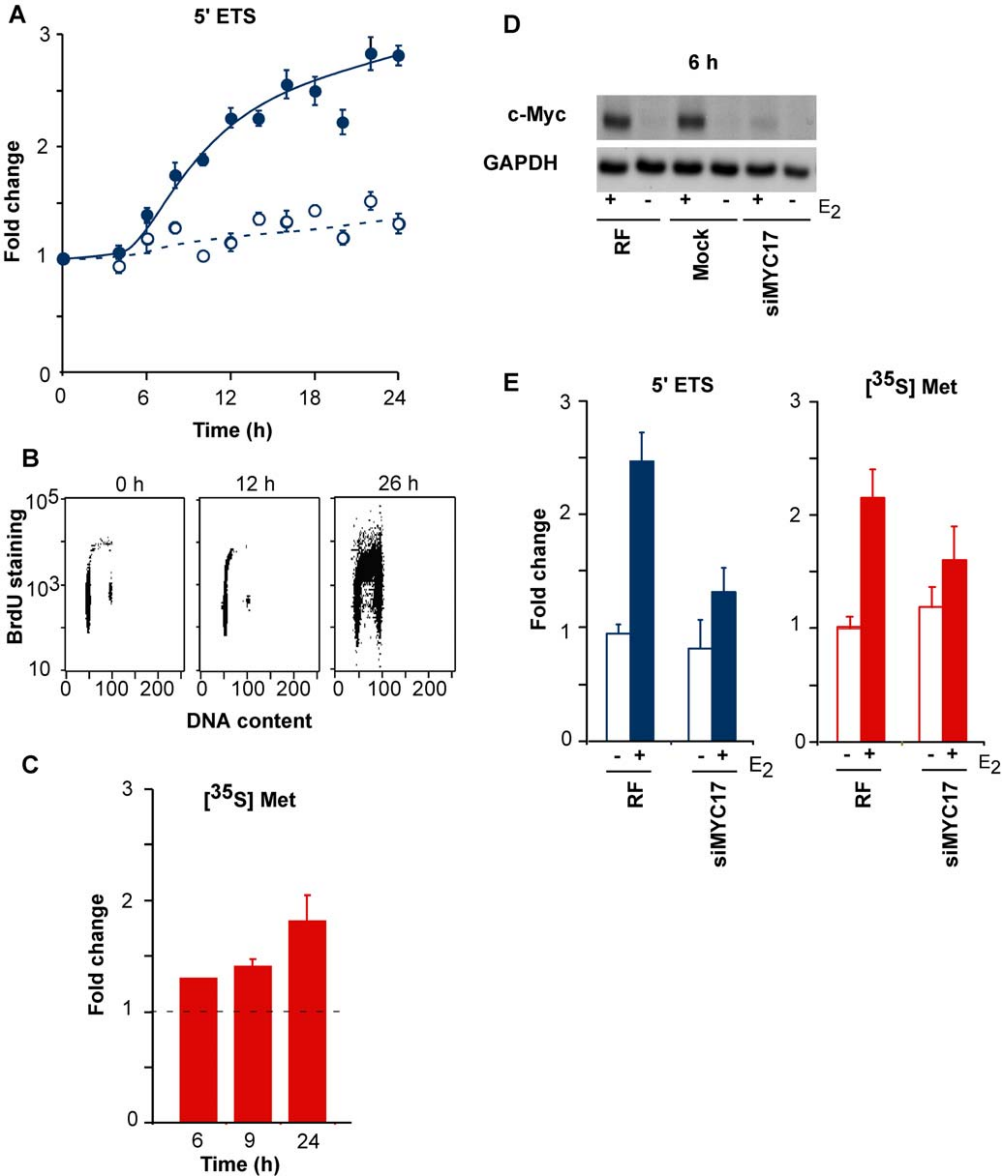
Effect of RNAi-mediated knockdown of c-Myc on cell growth after estrogen treatment. A, B: MCF-7 cells were growth arrested with 10 nM ICI 182780 for 48 h then treated with either 100 nM E_2_ or vehicle (ethanol) (A) Abundance of the 5′ETS of the 45S rRNA transcript was measured using qRT-PCR. Vehicle control, ○ open circles; estrogen, • filled circles. Data are mean±SEM of triplicate experiments. (B) Cells treated with E_2_ for the indicated times were additionally treated with BrdU 2 h before harvesting. BrdU content (immunofluorescence) and DNA content (propidium iodide staining) were measured using 2 parameter flow cytometry. (C) Overall protein synthesis was measured by [^35^S]-methionine incorporation at the indicated times after E_2_ treatment. Data are mean±range or SEM of 2–3 experiments. D, E: Cells were mock-transfected or transfected with 100 nM of control (RISC-free, RF,) or Myc siRNAs (siMYC17), treated with antiestrogen (ICI 182780) for 48 h, and then treated with 100 nM E_2_ or vehicle (ethanol). (D) Protein lysates were immunoblotted for c-Myc. (E) 5′ETS abundance (qRT-PCR) and protein synthesis ([^35^S]-methionine incorporation) were measured in the presence of control (RF) and c-Myc siRNAs (siMyc17) in duplicate experiments.

Cell growth is necessary but not sufficient for S phase entry and the two processes are closely co-ordinated [24]. Our pathway analysis indicated that estrogen may regulate cell growth principally via c-Myc. To test this prediction, we examined whether estrogen regulated cell growth in this model system. The first rate-limiting step in ribosome biogenesis is transcription of the 45S rRNA precursor, which is subsequently processed to yield rRNAs that are integral to the ribosomal subunits. The 5′ externally transcribed spacer (5′ETS) of the 45S rRNA began to increase in abundance 6–8 h after estrogen treatment (Fig. 6A), significantly preceding the initiation of DNA synthesis, which was first apparent after 12 h (Fig. 6B). Similarly, total protein synthesis measured by [^35^S]-methionine incorporation was increased by 6–9 h after estrogen treatment (Fig. 6C). These endpoints were then measured in antiestrogen-arrested MCF-7 human breast cancer cells stimulated with E_2_ in the presence ofsiMyc-17, which reduced the induction of c-Myc protein to less than 2-fold (Fig. 6D, see also Fig. 4). Under these conditions, the increase in 5′ETS levels was inhibited, as was estrogen induction of total protein synthesis (Fig. 6E). Thus, estrogen activates ribosome biogenesis and protein synthesis in a c-Myc-dependent manner, as predicted by our pathway analysis.

### Relationship between functional signatures and response to endocrine therapy

**Figure 7:**
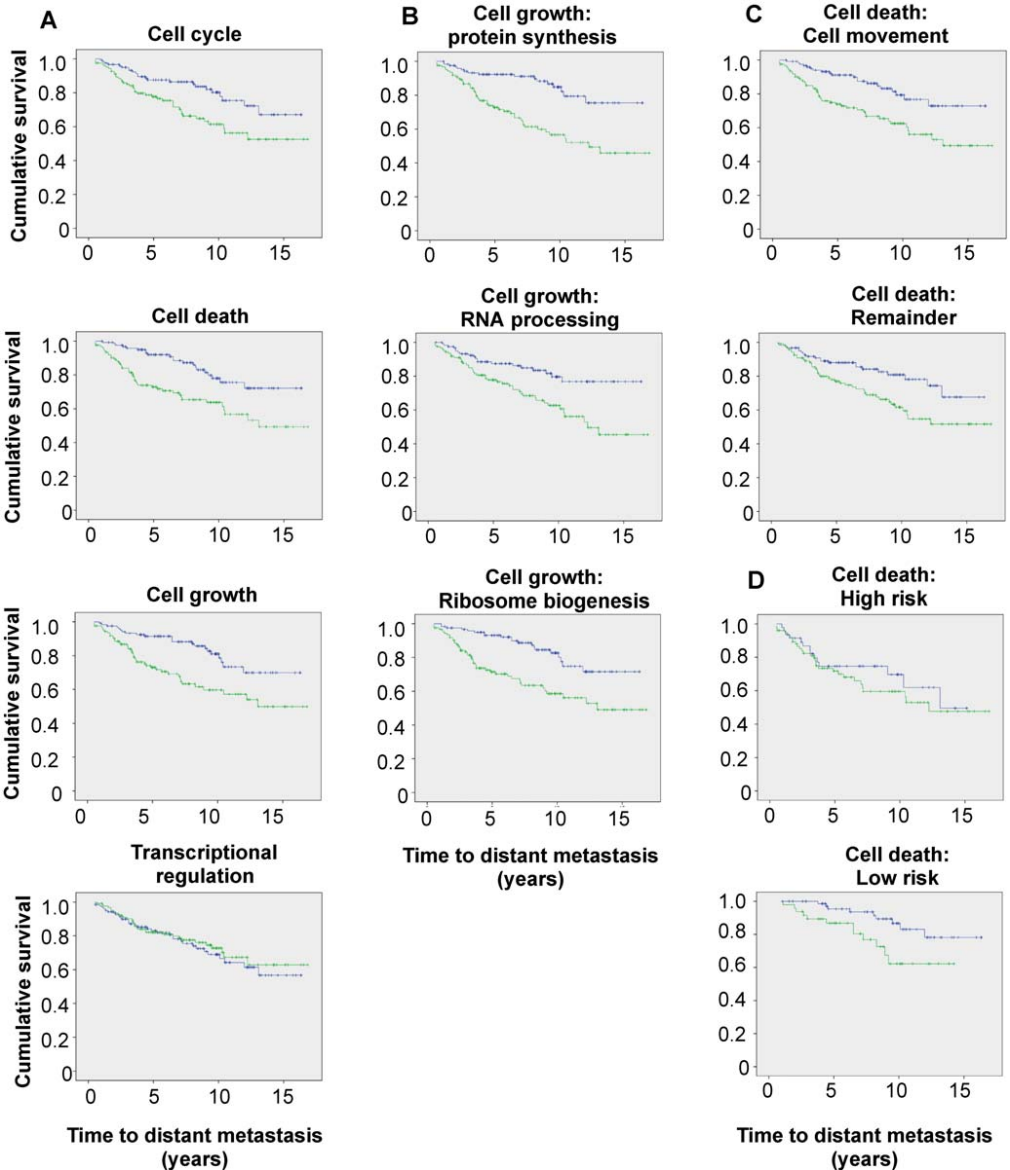
Relationship between estrogen-regulated gene signatures and response to tamoxifen therapy. Kaplan-Meier survival curves of the relationship between time to distant metastasis and the indicated gene signatures, dichotomised at the 50th percentile, in a cohort of 246 breast cancer patients treated with tamoxifen. (A) Signatures illustrated in Fig. 5. (B) Subsets of the ‘cell growth’ signature. (C) Subsets of the ‘cell death’ signature. (D) Patients were sequentially stratified using the ‘cell death’ and ‘cell cycle’ signatures.

To determine whether the individual processes regulated by estrogen might have different impacts on the response to endocrine therapy, we examined the relationship between the estrogen-regulated gene signatures and breast cancer patient outcome using transcript profiles generated from a population of 246 women with ER-positive breast cancer who received tamoxifen as their only adjuvant systemic therapy [11]. A semi-supervised principal component method [25] was used to assess the ability of each signature to predict time to development of distant metastasis. The ‘cell cycle’, ‘cell death’ and ‘cell growth’ signatures were all prognostic (Table 2, Fig. 7A), but the ‘transcriptional regulation’ signature was not, although it contained some genes that were individually significant predictors of outcome (for example, *CCND1* and *NCOA1/SRC-1*). *MYC* was not prognostic as a continuous variable (*P* = 0.372), and its inclusion in either the cell cycle or cell growth signatures did not influence their predictive ability. The three signatures that were significant remained predictive in multivariate models against standard clinicopathological parameters i.e. patient age, tumor grade, tumor size and lymph node status, whether analysed using the interquartile range (Table 3, models 1–3) or when treated as continuous variables (Cell cycle *P* = 0.001, HR 1.016, 95% CI 1.007–1.025; cell death *P* = 0.0001, HR 1.022, 95% CI 1.011–1.033; cell growth *P* = 0.002, HR 1.015, 95% CI 1.005–1.024). Since the ‘cell cycle’ and ‘cell growth’ signatures were strongly correlated with tumor grade, grade was omitted from the models for these signatures. To determine if the gene signatures gave prognostic information additional to the clinical variables described above, we generated a predictor based on a principal components analysis using the clinical variables, and then developed additional models using both the clinical variables and the individual gene signatures. Using this alternative approach, each signature still added significantly to the risk prediction using the clinical covariates alone (Cell cycle *P* = 0.002; cell death *P* = 0.05; cell growth *P* = 0.04).

**Table 2:** Analysis of functional signatures in tamoxifen-treated patients.

Signature	Log rank P
Cell cycle	**0.036**
Cell death	**0.004**
Cell movement	**0.014**
Remainder	0.076
Cell growth	**0.01**
RNA processing	**0.03**
Ribosome biogenesis	**0.001**
Protein synthesis	**0.002**
Transcriptional regulation	0.85

The log-rank p was generated after 500 permutations.

**Table 3:** Cox regression analysis.

Variable	HR	95%CI	*P*
**Model 1**			
Cell cycle	4.028	1.816–8.937	**0.001**
Patient age	0.833	0.283–2.449	0.740
Tumor size	1.948	1.129–3.361	**0.017**
Nodal status	1.274	0.729–2.226	0.396
**Model 2**			
Cell death	6.096	2.013–18.459	**0.001**
Patient age	0.902	0.267–3.049	0.868
Grade	1.011	0.636–1.608	0.962
Tumor size	1.988	1.059–3.734	**0.033**
Nodal status	1.323	0.718–2.439	0.370
**Model 3**			
Growth	3.690	1.561–8.726	**0.003**
Patient age	0.959	0.333–2.761	0.937
Tumor size	1.875	1.081–3.252	**0.025**
Nodal status	1.411	0.819–2.432	0.215
**Model 4**			
Cell cycle	2.401	0.993–5.804	0.052
Cell death	3.006	1.258–7.185	**0.013**
Cell growth	2.097	0.798–5.514	0.133
**Model 5**			
Cell death	3.344	1.400–7.987	**0.007**
Cell cycle	2.785	1.203–6.446	**0.038**
Patient age	0.672	0.266–2.351	0.790
Tumor size	1.881	1.088–3.254	**0.024**
Nodal status	2.430	0.772–2.430	0.282
**Model 6**			
Cell death	3.421	1.450–8.075	**0.005**
Cell growth	2.469	1.003–6.081	**0.049**
Patient age	0.876	0.299–2.564	0.809
Tumor size	1.846	1.062–3.210	**0.030**
Nodal status	1.429	0.821–2.489	0.207

For each signature the interquartile HR is shown i.e. highest vs. lowest quartile. Grade is treated as a continuous variable (1 vs. 2 vs. 3) while tumor size (≤2 cm vs. >2 cm), age (≤50 vs. >50 yrs), and nodal status (negative vs. positive) are binary variables.

To gain possible insights into the contribution of components of the processes represented by each signature, we further divided the signatures. When the ‘cell growth’ signature was subdivided into components representing RNA processing, ribosome biogenesis and protein synthesis, each was individually prognostic (Table 2, Fig. 7B). Because the ‘cell death’ signature contained some genes that also had roles in processes that might be expected to affect metastasis, i.e. cell migration and invasion, we assessed the prognostic ability of genes with a ‘cell movement’ annotation compared with the remainder of the signature. The ‘cell movement’ subgroup was predictive (Table 2, Fig. 7C). However, although for the remainder of the ‘cell death’ signature the high risk and low risk groups diverged, this was not statistically significant (Table 2, Fig. 7C).

The question of whether these signatures identified distinct groups of patients is important in relation to both their potential clinical utility and a better understanding of the underlying biological mechanisms. There were strong correlations between the cell cycle and cell growth signatures (r = 0.653, p<0.01), but weaker associations between cell cycle and cell death (r = 0.326, p<0.01), or cell growth and cell death (r = 0.385, p<0.01). Consistent with the idea that the cell death signature might identify a distinct subset of patients, the discordance between classification on the basis of the cell cycle or cell death signatures was 42%, compared with 27% between the cell cycle and cell growth signatures. Furthermore, patients classified as low risk based on the ‘cell death’ signature, but not those classified as high risk on this basis, could be separated into groups of differing outcome on the basis of the ‘cell cycle’ signature (p = 0.015 and 0.67, respectively; Fig. 7D). Finally, multivariate analysis comparing all three signatures as continuous variables revealed that the cell death signature was predictive independent of the other two signatures whether analysed using the interquartile range (Table 3, Model 4), or as continuous variables (Cell cycle *P* = 0.058, HR 1.010, 95% CI 1.000–1.021; cell death *P* = 0.009, HR 1.013, 95% CI 1.003–1.023; cell growth *P* = 0.287, HR 1.006, 95% CI 0.995–1.017). In models including either ‘cell cycle’ or ‘cell growth’ against ‘cell death’ and the clinicopathological variables, all remained predictive when analysed using the interquartile range (Table 3, Models 5&6), although ‘cell growth’ did not when treated as a continuous variable (Cell death vs cell cycle: cell cycle *P* = 0.006, HR 1.013, 95% CI 1.004–1.023; cell death *P* = 0.038, HR 1.010, 95% CI 1.001–1.020. Cell death vs. cell growth: cell death *P* = 0.005, HR 1.014, 95% CI 1.004–1.024; cell growth *P* = 0.081, HR 1.009, 95% CI 0.999–1.019). The ‘cell death’ and ‘cell cycle’ signatures therefore apparently confer independent prognostic information.

## Discussion

Estrogen action is necessary both for the normal development of the female reproductive organs, including the mammary gland, and for the development and proliferation of a majority of human breast cancers. However, understanding the mechanisms underlying the physiological effects of this important hormone and how their deregulation impacts on sensitivity to therapies directed at the ER remains a significant challenge.

### Role of c-Myc in estrogen action

By analysis based on functional annotation of acutely estrogen-regulated genes we have identified gene signatures that encompass four different aspects of estrogen action, and contain different proportions of c-Myc-responsive genes: cell cycle control, cell growth, cell death and transcriptional regulation. The proportion of human protein-coding genes that is c-Myc-responsive is estimated at 10–15% and many non-coding RNAs are also regulated by c-Myc [26]. In addition, c-Myc and ERα binding sites co-localise near the transcription start site of a subset of estrogen-responsive genes [27]. Nonetheless, the observation that as many as half of estrogen-responsive genes are also c-Myc-responsive is striking and unexpected, and argues strongly for a critical role for c-Myc in estrogen action. This was revealed by our focus on the acute effects of E_2_ and use of an experimental model designed to maximise sensitivity to the ability of E_2_ to promote the G_1_-S phase transition, in contrast with previous analyses which have often been undertaken over longer timeframes or in experimental models with less sensitivity to effects on proliferation. The different contributions of c-Myc-responsive genes to the different estrogen-responsive signatures indicates that the role of c-Myc may be specific to some processes, rather than global.

The ‘cell cycle’ network presented here integrates upstream cell cycle regulatory molecules with those more directly involved in DNA replication i.e. PCNA, Cdc6, and the MCMs. Recent evidence that c-Myc is associated with the pre-replication complex suggests one means by which estrogen could regulate DNA replication [28]. However, c-Myc regulates the number of replication origins rather than the rate of replication fork movement [28], while estrogen increases the rate of replication fork movement [29]. Since many of these DNA replication genes are E2F-responsive, estrogen stimulation of E2F activity as cells progress through G_1_ phase [30], [31] provides a likely mechanism for their activation. Interestingly, *CDKN1A*, the gene encoding p21^Waf1/Cip1^, is a prominent hub linking many of the genes within the ‘cell cycle’ signature, consistent with studies identifying p21^Waf1/Cip1^ as an important effector of c-Myc action on the cell cycle in estrogen-treated cells [13], [19]. Had this not already been known, our analysis would have suggested p21^Waf1/Cip1^ as a strong candidate for further investigation, highlighting the ability of these functional pathways to provide mechanistic insights and suggesting that some of the hubs in other networks merit further investigation as mediators of estrogen action.

Like the ‘cell cycle’ signature, the ‘transcriptional regulation’ signature contained approximately equal proportions of c-Myc-regulated genes and genes unresponsive to c-Myc activation. Prominent in the ‘transcriptional regulation’ network are a number of nuclear receptor coregulators (the coactivators NCOA1/SRC-1/RIP160, NCOA2/SRC-2/GRIP1, NCOA3/AIB1/SRC-3, NCOA7, ARNT, ARNT2 and the corepressor NRIP1/RP140), which play a central role in transcriptional activation by members of the nuclear receptor superfamily [32] and which were regulated in a manner consistent with the well-known ligand-activated downregulation of estrogen receptor signaling [33]. Interestingly, NCOA1, ARNT and ARNT2 and one of two probsets for NCOA3 were also significantly downregulated by c-Myc, suggesting that c-Myc-mediated repression may also contribute to this response.

Estrogen regulates both RNA and protein synthesis, an important physiological response that was the focus of much early work on estrogen action [34]. However, the molecular mechanisms for estrogen effects on cell growth remain largely unexplored. We show here that almost all of the acutely estrogen-regulated genes with roles in cell growth are also c-Myc targets, and that estrogen activation of rRNA synthesis and protein synthesis depends on c-Myc. The idea that estrogen regulates cell growth via c-Myc is supported by evidence that estrogen induction of c-Myc in the rodent uterus is not prevented by progesterone, which inhibits DNA synthesis but not growth [35], and the known role of c-Myc in regulating cell growth in mammalian cells [24], [36].

Like the ‘cell cycle’ network, the ‘cell death’ network contains both effectors and upstream regulators. Bcl-2 acts as a hub connecting many of the effectors, consistent with the well-documented role of Bcl-2 as a mediator of the anti-apoptotic effects of estrogen [37], [38]. The ‘cell death’ signature also contains components of survival signaling pathways, e.g. receptor tyrosine kinases (IGF1R and Ret) and their effectors (IRS1, Jak1, Jak2) that are increased in response to estrogen treatment. The implications of transcriptional regulation of these signaling pathways has been much less well-studied than their regulation by protein-protein interactions and phosphorylation, but a co-ordinate increase in expression is likely to result in enhanced survival signaling. Estrogen suppression of apoptosis resulting from growth factor deprivation is c-Myc dependent [39] but genes regulated by c-Myc were under-represented in the ‘cell death’ signature. However, our experimental model, i.e. cells cultured in the presence of serum and insulin, is rich in survival factors, and the ability of estrogen to enhance survival signaling may further oppose the ability of c-Myc to promote apoptosis in this model.

### Deregulation of estrogen action and endocrine resistance

An association between increased breast cancer proliferation and poor outcome in response to endocrine therapies is clearly apparent from studies measuring both individual markers of proliferation (e.g. Ki67) and gene signatures associated with reduced survival [10], [40]–[42]. The particularly poor outcome of highly proliferative ER-negative breast cancers and the association between signatures containing prominent proliferation-related components, for example the genomic grade signature [11], and poor outcome in both untreated and tamoxifen-treated ER+ve breast cancers [10], raises the question of whether increased proliferation *per se* is a marker of an adverse prognosis, or whether there are aspects of loss of proliferative control that affect response to individual therapies. Whether the signatures identified here are specifically predictive of response to tamoxifen or might also be associated with poor response to other therapies remains to be determined. However, in contrast with previous analyses in breast cancer, we have distinguished cell cycle/cell growth, and cell survival signatures that are independent predictors of outcome in tamoxifen-treated patients.

A recent analysis of ‘molecular concepts’ associated with progression of prostate cancer identified increased protein synthesis and enrichment at chromosome 8q, which includes *MYC* (8q24), as features distinguishing the precursor lesion prostatic intraepithelial neoplasia (PIN) from benign epithelium [43]. The proliferation signature was distinct from the protein synthesis network, and although both increased during disease progression, they did so at different stages [43], consistent with the idea that enhanced cell cycle progression and enhanced cell growth may reflect different initiating events. In our analysis c-Myc-responsive genes predominated in the ‘cell growth’ signature, which contains many of the most strongly c-Myc-regulated genes. The ‘cell growth’ signature may therefore be a surrogate measure of deregulated c-Myc expression that identifies a subset of proliferative, endocrine-resistant breast cancers with distinct biology.

The poor outcome associated with the ‘cell growth’ signature may reflect a specific resistance to endocrine therapies associated with deregulation of c-Myc. However, the ‘wound signature’, which is induced by co-ordinate amplification of *MYC* and *CSN5/JAB1/COPS5* [44], is predictive of a poor outcome in a cohort of patients with ER-positive cancers who were more commonly treated with chemotherapy than endocrine therapy [45], suggesting that deregulation of c-Myc may result in resistance to multiple therapies.

The well-established role of estrogen in promoting cell survival suggests that increased apoptosis might be associated with a better clinical response to endocrine therapies. However, clinical studies addressing this question have often revealed conflicting data, perhaps because of inherent difficulties in accurately monitoring the dynamics of apoptosis *in vivo* [reviewed in 46]. As found by another study identifying genes differentially expressed in tamoxifen-sensitive and -insensitive breast cancer [47], the adverse outcome predicted by the ‘cell death’ signature was associated with both pro-apoptotic and anti-apoptotic changes in gene expression. Consequently the likely effect of the observed changes in expression within the ‘cell death’ signature is not clear. The dual role of a subset of genes within the signature in regulating invasion and motility provides another mechanism whereby their deregulation may impair response to endocrine therapies. For example, Bcl-2, which has functional interactions with many other genes within the signature, has been implicated in estrogen regulation of invasion downstream of RelB [48].

In summary, the main findings of this work are the predominance of c-Myc as a target of estrogen action, its specific role as a mediator of estrogen effects on cell growth, and the ability of functionally-based gene signatures to stratify patients treated with tamoxifen into groups with differing outcome, potentially identifying distinct mechanisms of tamoxifen resistance. This provides an opportunity to identify new therapeutic options for endocrine-resistant breast cancer.

## Materials and Methods

### Plasmid construction and transfection

The plasmids pΔMT and pΔMT-Myc, which contain a metal-inducible metallothionein promoter [49] have been previously described [13]. pΔMT-c-Zip was constructed using the mouse c-Zip cDNA subcloned from KS c-Zip [50]. MCF-7 cells were transfected with either pΔMT, pΔMT-c-Myc or pΔMT-c-Zip together with a plasmid containing a selectable marker (puromycin). Multiple individual puromycin-resistant colonies (10–20 for each construct) were isolated, expanded and characterised.

### Cell culture, DNA flow cytometry and measurement of protein synthesis

MCF-7 cells were maintained as previously described [51]. Stock solutions of the pure antiestrogen ICI 182780 {7α-[9-(4,4,5,5-pentafluoropentyl-sulfinyl)nonyl]estra-1,3,5(10)-triene-3,17β−diol} and the steroid estradiol (17β-estradiol) were prepared as described previously [52]. Stocks of Nocodazole {methyl-[5-(2-thienyl-carbonyl)-1H-benzimidazol-2-yl] carbamate} were prepared in DMSO and used at a final concentration of 50 ng/ml.

Exponentially proliferating cells were growth arrested by pretreatment for 48 h with the steroidal antiestrogen ICI 182780 (10 nM) and then treated with either 100 nM E_2_ or 65 µM Zn (as ZnSO_4_) as previously described [13]. DNA analysis by flow cytometry was as previously described [13]. To measure protein synthesis MCF-7 cells were labeled with 45 μCi [^35^S]-methionine for 20 mins as previously described [53].

### Myc siRNA

siRNAs (siMyc-17 (D-003282-17-0050), non-targeting control 2 (D-001210-02-20) and RISC-Free siRNA (D-001220-01-20)) were purchased from Dharmacon (Lafayette, Colorado, USA) and transfected at 50 or 100 nM using Lipofectamine 2000 (Invitrogen). Transfection with fluorescinated siRNAs showed that >98% of target cells were transfected. The siRNA/transfection mix was removed 24 h after transfection and replaced with fresh RPMI medium containing ICI 182780 (10 nM). After a further 48 h the cells were treated with vehicle (ethanol) or E_2_ (17-β estradiol, 100 nM).

### Western blot analysis

Cell lysates were prepared and immunoblotted as previously described [52]. The following primary mouse monoclonal antibodies were used: cyclin D1 (DSC-6, Novocastra, Laboratories Ltd, Newcastle-upon-Tyne, UK), β-actin (AC-15, Sigma, St Louis, MO, USA), p21 (610233; BD Transduction Laboratories, Lexington, KY, USA), p27 (610241, BD Transduction Laboratories, Lexington, KY, USA), pRB (554136, BD Pharmigen, San Diego, CA, USA), c-Myc (9E10, Santa Cruz Biotechnology Inc, Santa Cruz, CA, USA). The following primary rabbit polyclonal antibodies were used: cyclin A (C-19), and c-Myc (C-19) (Santa Cruz Biotechnology).

### Quantitative Real-Time PCR

Total RNA was isolated using the RNAeasy kit (Qiagen) from E_2_ or vehicle and Zn-treated cells and reverse-transcribed using the Reverse Transcription System (Promega, NSW, Australia). Real-time PCR was performed using an ABI Prism 7900HT Sequence Detection System using inventoried Taq-Man probes (Applied Biosystems, Foster City, CA, USA). *GAPDH* and *RPLPO* were used as internal controls.

### Transcript profiling and microarray data analysis

RNA was collected in three independent experiments, each including parental cells treated with E_2_ or ethanol, zinc-treated pΔMT-c-Myc cells, zinc-treated pΔMT-c-Zip cells and zinc-treated empty vector (pΔMT) cells. Cells were arrested for 48 h with 10 nM ICI 182780 and then treated for 6 h with either 100 nM E_2_ or ethanol vehicle, or 75 µM zinc for the stably transfected cell lines. Target probes were prepared and hybridised to Affymetrix Human Genome U133 Plus 2.0 oligonucleotide microarrays (Millennium Science, Box Hill, Vic, Australia) according to the manufacturer's instructions. The microarray data are available through the Gene Expression Omnibus (GEO) database (accession number GSE11791).

Quality of the arrays was assessed using histograms of probe intensity, RNA degradation plots and Affymetrix-style quality control measures generated using functions within the *affy* and *simpleaffy* package of Bioconductor [54]. The arrays showed similar ‘gamma’ shaped distributions of probeset intensities, and had scaling factors of 0.995–2.048 units, confirming their high quality and suitability for batch normalization. Normalization of the arrays was performed using the RMA algorithm, as implemented in the *affy* package of Bioconductor and using the default options of RMA (with background correction, quantile normalization, and log transformation).

After normalisation probesets with intensity >100 in any of the experimental conditions were analysed using Bayesian linear modelling in the *limma* package [55], with replicate and treatment as fixed effects. Penalized t-statistics from these comparisons were generated by Benjamini and Yekutieli adjustment for multiple-hypothesis comparisons [56] using the *multtest* package and probes displaying significant differential expression (adjusted p<0.01) were identified and compared with databases of estrogen-responsive genes ((ERGDB, http://research.i2r.a-star.edu.sg/promoter/Ergdb-v11/, [16]), c-Myc targets (http://www.myc-cancer-gene.org, [17]), and nucleolar proteins (http://www.lamondlab.com/NOPdb/, [22] current in November 2005. Pathway analysis used Onto-Express (http://vortex.cs.wayne.edu/index.htm, [20]), Ingenuity Pathways Analysis (Ingenuity Systems, Redwood City, CA, http://www.ingenuity.com), and HiMAP (www.himap.org [21]. Gene ontology and functional annotation is presented for only those categories containing ≥3 genes and with an adjusted *P* value <0.01.

### Clinical microarray data

The clinical dataset used for analyzing the relationship between the functional gene signatures and response to endocrine therapy consisted of 246 primary breast cancer samples. The demographics and methods have been previously described [11] and the raw data are available at the GEO database (accession number GSE6532). All samples were required to be estrogen and/or progesterone receptor positive by ligand-binding assay and had received tamoxifen monotherapy only in the adjuvant setting. The cut-off value for classification of patients as ER-positive or -negative was 10 fmol per mg protein. The primary endpoint used for generating the classifiers was the first distant metastatic event as survival can be confounded by local recurrence and treatments given at relapse.

Data analyses were performed using version 3.5 of BRB ArrayTools (http://linus.nci.nih.gov/BRB-ArrayTools.html). The survival analyses were performed using the ‘survival risk prediction tool’ where the survival risk groups are constructed using a supervised principal component method [25]. All genes from each of the functional networks that were present on the U133A Affymetrix microarrays used for analysis of gene expression in tamoxifen-treated patients were entered to generate the classifier. Two principal components, 10-fold cross-validation and a binary cut-off using the 50^th^ percentile were used to generate two prognostic groups. The log-rank *P* value for the Kaplan-Meier curve was generated after 500 permutations. To determine the predictive accuracy of each of the functional gene signatures compared with the clinical prognostic variables alone, two models were created for each signature—one with clinical covariates only and one with the clinical covariates and the gene signature. The cross-validated Kaplan-Meier curves and log-rank statistics for these models were generated after 500 permutations and the *P* value measures whether the expression data significantly adds to predictive accuracy compared with the clinical factors alone. This approach is preferable to a multivariate model which only compares prognostic effects and can be unstable due to multi-colinearity between variables in the model and random fluctuations in the data. Multivariate Cox regression analyses were performed using SPSS statistical software package (SPSS Inc. Chicago, IL) version 13.0. Each gene signature and tumor grade (1 vs. 2 vs. 3) were treated as continuous variables, while tumor size (≤2 cm vs. >2 cm), patient age (≤50 vs. >50 yrs) and nodal status (positive vs. negative) were treated as binary variables.

## Supporting Information

Table S1 Estrogen-regulated genes
(0.46 MB XLS)

Table S2 Estrogen-regulated gene signatures.
(0.13 MB XLS)

Table S3 Ingenuity annotation of gene signatures
(0.17 MB XLS)
